# Concurrent and Predictive Relationships Between Compulsive Internet Use and Substance Use: Findings from Vocational High School Students in China and the USA

**DOI:** 10.3390/ijerph9030660

**Published:** 2012-02-23

**Authors:** Ping Sun, Carl Anderson Johnson, Paula Palmer, Thalida E. Arpawong, Jennifer B. Unger, Bin Xie, Louise A. Rohrbach, Donna Spruijt-Metz, Steve Sussman

**Affiliations:** 1 Institute for Health Promotion and Disease Prevention Research, Department of Preventive Medicine, Keck School of Medicine, University of Southern California, 2001 N. Soto Street, Los Angeles, CA 90032, USA; Email: arpawong@usc.edu (T.E.A.); unger@usc.edu (J.B.U.); rohrbac@usc.edu (L.A.R.); dmetz@usc.edu (D.S.-M.); ssussma@usc.edu (S.S.); 2 School of Community & Global Health, Claremont Graduate University, 160 East 10th Street, Claremont, CA 91711, USA; Email: andy.johnson@cgu.edu (C.A.J.); paula.palmer@cgu.edu (P.P.); bin.xie@cgu.edu (B.X.)

**Keywords:** compulsive internet use, internet addiction, youth, cigarette smoking, binge drinking, addiction syndrome, addiction specificity

## Abstract

*Purpose:* Compulsive Internet Use (CIU) has increasingly become an area of research among process addictions. Largely based on data from cross-sectional studies, a positive association between CIU and substance use has previously been reported. This study presents gender and country-specific longitudinal findings on the relationships between CIU and substance use. *Methods:* Data were drawn from youth attending non-conventional high schools, recruited into two similarly implemented trials conducted in China and the USA. The Chinese sample included 1,761 students (49% male); the US sample included 1,182 students (57% male) with over half (65%) of the US youth being of Hispanic ethnicity. Path analyses were applied to detect the concurrent and predictive relationships between baseline and one-year follow-up measures of CIU level, 30-day cigarette smoking, and 30-day binge drinking. *Results:* (1) CIU was not positively related with substance use at baseline. (2) There was a positive predictive relationship between baseline CIU and change in substance use among female, but not male students. (3) Relationships between concurrent changes in CIU and substance use were also found among female, but not male students. (4) Baseline substance use did not predict an increase in CIU from baseline to 1-year follow-up. *Conclusions:* While CIU was found to be related to substance use, the relationship was not consistently positive. More longitudinal studies with better measures for Internet Addiction are needed to ascertain the detailed relationship between Internet addiction and substance use.

## 1. Introduction

Although it is still debatable whether Internet Addiction (IA) should be labeled as an addictive disorder [1,2,3,4], the detrimental effects of IA have been widely observed and documented [3,5,6]. Furthermore, while IA may not be as widely recognized as an addictive behavior in the United States (US), the harmful effect of IA has been recently demonstrated in China and other Asian countries [7]. It has been estimated that 14.1% of adolescents—roughly 20 million people—met the diagnostic criteria for IA in China, and the prevalence of IA is highest among the 18–23 year old age group [8]. National laws are being implemented in China with the aim of reducing IA among youth, especially through uncontrolled use in cyber cafés [3]. To effectively help those who are in need of assistance, evidence based school-level or student-level IA intervention methods are being requested by parents and health educators [9]. 

In order to develop evidence-based programs aimed at controlling IA, it is essential to fully understand its etiological pathway and patterns of psychiatric and behavioral co-morbidities. IA has been found to be a co-morbidity with psychiatric symptoms [10,11] and other risk factors [12] of substance use, or substance use dependencies [13]. Research suggests that addiction to internet use is similar to addiction to other compulsive behaviors such as substance use, binge eating [14] (page 479), compulsive gambling [15], excessive shopping [16], and compulsive sex behavior [17]; they are all symptoms of maladaptive process addictions. A syndrome hypothesis of addiction [18] posits that addiction may not be inextricably specific to a particular substance or behavior. For example, various addictions are found to co-exist [19] with addicts often alternating between substances [20]. Furthermore, it is common that addicts may switch from substance use to a compulsive behavior [21]. Thus, with respect to uncovering etiological patterns of IA as a current and lifetime co-morbidity, it will be important to examine the complete concurrent and longitudinal relationships between IA and substance use.

Currently, available data that relates IA with other addictive behaviors (substance abuse) is very limited [22]. Analyses of cross-sectional data have revealed that IA was positively related to substance use among youth [23,24,25,26,27]. IA was also positively correlated with certain risk factors of substance use behavior (e.g., intention to use drugs, pro-drug use attitudes, perceived social norm of drug use) [26]. However, there is a dearth of longitudinal studies that contribute to understanding not only the cross-sectional concurrence between IA and substance use, but also the other hypothesized relationships over time. 

Research focusing on IA has been much more prominent in Asian countries [3], which may reflect the fact that consequences of IA are perceived as more harmful in Asian countries than in the western countries [3]. Results of one study suggest that there are differences in IA by race [28]; thus, it will be important to compare findings from similar studies conducted in both Eastern and Western countries. 

In general, hypotheses regarding the comprehensive nature of the relationships between IA and substance use may include the following: (1) IA is a causal risk factor for substance use; (2) substance use is a causal risk factor for IA; (3) IA and substance use reinforce each other and thus have a reciprocal relationship; and (4) concurrent relationships exist between measures of IA and substance use. If there are no relationships found to support hypotheses 1, 2, or 3; but there is a relationship that supports hypothesis 4, there would be no true causal relationship between IA and substance use; rather the observed concurrent relationships between the cross-sectional measures, or between parallel changes during the same time span could be spurious (*i.e.*, caused by a third factor). 

There is currently a lack of universal diagnostic criteria for IA. Viewing IA as an impulse-control disorder that does not involve an intoxicant, researchers consider IA as a multi-dimensional disorder [7,29,30]. Together with other aspects of IA, compulsive internet use (CIU) is a commonly accepted dimension of IA. The relationships between CIU, cigarette smoking, and binge drinking are the focus of this study. 

Objectives of this study are uniquely comprehensive in that we will examine all four hypothesized relationships between CIU and substance use using longitudinal data. Namely, we will depict the possible concurrent and reciprocal predictive relationships between CIU, cigarette smoking, and binge drinking behaviors among high-risk youth. The concurrent relationships investigated will include both the cross-sectional (baseline with baseline) and longitudinal ones (e.g., how the change in CIU from baseline to follow-up correlate with the change in cigarette smoking from baseline to follow-up). All change scores will be calculated as the value at 1-year follow-up survey minus the value at baseline. All possible bidirectional relationships will be investigated such that baseline behaviors will be used to predict change in behavior from baseline to one-year follow-up. All analyses will be conducted separately in US and Chinese samples, and separately for each gender. Therefore, analyses will also be able to distinguish the possible differences in the hypothesized relationships between the two countries, and by gender. 

## 2. Methods: Study Design and Sample Description

Data for the analyses were drawn from two similar school-based longitudinal trials, one conducted in Chengdu, China and the other in Los Angeles, California, USA. The study in China, the “Healthy Lives, Successful Lives” (HLSL) trial, was a two-condition controlled intervention conducted to test the impact of a 10 lesson (45 minutes each) intervention on tobacco use and alcohol use among high school students over two years. The US study, a “Towards No Drug Abuse” (TND) trial, was a three-condition controlled intervention implemented to test the impact of TND programming *vs.* “a standard care control program” over three years. Each study employed a design where program interventions were applied between a pre-intervention baseline measure and several post-intervention follow-up measures. Data for these analyses were drawn from measures collected at two time points: baseline and one-year follow-up. The US study included 1,182 students recruited from 24 continuation high schools (CHS) in California. The China study included 1,761 10th grade students from 12 vocational high schools (VHS). Both types of schools—CHS in California and VHS in China—are high schools that differ from regular high schools because of their student enrollment methods. In California, students who are unable to remain in the regular high school system due to functional reasons (e.g., did not fulfill credit requirement for graduation, consistent use of substances, truancy) are transferred to CHS. In China, students who are unable to perform academically on the high school admittance test or are enduring household economic challenges typically end up in VHS. The details of the study design and data collection procedures for these two studies were introduced elsewhere [31,32]. All study procedures, including informed consent, for both studies were approved by the University of Southern California’s Institutional Review Board (IRB). Procedures for the China study gained additional approvals from the local IRB in China. 

The average age (years, mean ± std) was 16.8 ± 0.93 and 15.9 ± 0.76 for the students in the TND, and HLSL study, respectively. The TND study included slightly more male students than the HLSL study (56.6% *vs.* 49.1%). In TND, more than half of the students were Hispanic (64.9%), with 11.7% being Non-Hispanic White, 4.3% African-American, 13.0% Mixed ethnicity, and 6.1% other Ethnicity. In the HLSL study, almost all youth were of Han ethnicity.

## 3. Measures

This paper focuses on the concurrent and predictive relationships between CIU, cigarette smoking, and binge drinking. The key analytical variables included CIU, 30-day cigarette use, 30-day binge drinking, and other covariates that are adjusted for in the analyses.

Various methods have been used to assess problematic internet use [7,29,33]. Our measure of CIU was based on the Davis Scale for problematic internet use [30,33]. In this scale, Davis proposed four dimensions with which to assess consequential CIU: diminished impulse control, loneliness/depression, social conformity, and distraction. Limited by the number of items that could be placed on the surveys, we chose to focus on assessing diminished impulse control with the four items that may be best suited for revealing shared pathways with other addictive behaviors (e.g., substance use [34,35]); we labeled the measure “Compulsive Internet Use” (CIU). The four items comprising CIU were “I use the Internet more than I ought to”, ”I usually stay on the Internet longer than I had planned”, “Even though there are times when I would like to, I can't cut down on my use of the Internet”, and “My use of the Internet sometimes seems beyond my control.” Response options were provided on an ordinal scale, including (1) Strongly disagree, (2) Disagree, (3) Neutral, (4) Agree, and (5) Strongly agree. The mean of all four items was used as a continuous measure of CIU. The CIU scale showed a high inter-item consistency for the four items with a Cronbach alpha of 0.84 and 0.80 among the Chinese and US sample, respectively. The test-re-test correlation for CIU between baseline and 2-month follow-up was 0.48 for Chinese and 0.61 for US youth; and between baseline and 1-year follow-up, it was 0.39 for Chinese and 0.48 for US youth.

Cigarette smoking and binge drinking in last 30 days were assessed slightly differently in TND and HLSL studies. In TND, cigarette smoking was assessed by the question, “How many times in the last month have you used cigarettes?”, with a 12-point scale response option, starting at “0 times”, increasing in intervals of 10 (e.g., “1–10 times”, “11–20 times”) with the last (12th) category being “over 100 times”; binge drinking was assessed by the question, “How many days have you had 5 or more alcoholic drinks within a 5 hour period over the last 30 days?” with a similar 12-point scale response option. In the HLSL study, cigarette smoking was assessed by the question “During the last 30 days, on how many days did you smoke cigarettes?”, with a 7-level answer option ranging from 1: “0 day”, 2: “1 or 2 days”, …, 6: “20–29 days”, to 7: “all 30 days”. The binge drinking behavior was assessed by asking the participants “During the past 30 days, on how many days did you have 5 or more drinks of alcohol in a row, that is, within a couple of hours?” with the 7-level answer options ranging from 1: “0 day”, 2: “1 day”,…, to 7: “20 or more days”. Dichotomous transformation of the substance use measures were applied such that any use on one or more days is coded as “Yes” for use (*i.e.*, presence of cigarette smoking or binge drinking in past 30 days).

To assess any differential relationship across gender, analyses were conducted separately for boys and girls. Analyses were adjusted for potential demographic and environmental confounders. The covariates for the Chinese sample included age, students’ birthplace (urban *vs.* rural or suburban), parents’ education (highest among both parents with seven levels ranging from 1: ‘lower than elementary school’ to 7: ‘4-year or higher’), allowance (a 10-level answer for weekly amount, ranging from 1: ‘none’, to 9: ‘more than 90 Yuan’), self-reported academic performance (5-levels, ranging from 1: ‘F or below’, to 5: ‘A or above’), a dummy variable indicating intervention status (program *vs.* control), and a propensity score for attrition. Covariates for the US sample included age, ethnicity, a dummy variable indicating intervention status (program *vs.* control), and a propensity score for attrition. The purpose for including a propensity score for attrition as a covariate is to adjust for the potential bias introduced by non-random attrition at follow-up [36]. The propensity score was generated by using logistic regression analysis, such that selected baseline measures were used to predict the dichotomous attrition status at one-year follow-up. A continuous propensity score for attrition was then calculated from the estimated formula. 

## 4. Statistical Modeling

Due to the multi-level sampling method employed in both studies (targeted school, and students within the targeted schools), multi-level random coefficients modeling was applied to the analysis, in which a school-level random effect was assumed in the model. Structural equation modeling was employed to assess all concurrent and predictive relationships in one SEM model. Mplus [37] was used to construct the SEM modeling, which estimates the independent relationships among the three behaviors targeted. All estimates from the model could then be viewed as the relationships between the residuals of the variance, after adjusting for all other relationships in the pathway as well as the contributions from covariates. Standardized coefficients for the relationships (partial correlations) were reported for the path diagrams. Additional analyses were also conducted to examine if the relationships were moderated by intervention status, such that interaction terms were computed and modeled in the analyses. 

## 5. Results

The mean (±std) for CIU was 2.27 (±0.92), 2.71 (±1.02), 2.45 (±0.96), and 2.32 (±0.93) among the Chinese female, Chinese male, US female, and US male students, respectively. While this continuous measure is not a suitable estimate of CIU prevalence, if assuming that an average score of 4 (“Agree” or “Strongly agree” with the CIU statements) is the cut-off for determining the binary status of having CIU problem, the prevalence of CIU would be 5.8%, 15.7%, 9.7%, and 7.3% among the Chinese female, Chinese male, US female, and US male students, respectively. Other studies in China have estimated the prevalence of Internet addiction among Chinese youth to be between 5% and 15% [7,8,38], which is consistent with our estimations for the Chinese youth. 

The prevalence of 30-day cigarette smoking was 13.7%, 46.1%, 36.6%, and 46.3% among the Chinese females, Chinese males, US females, and US males, respectively. The prevalence for binge drinking was 3.7%, 10.9%, 34.2%, and 38.8% among the Chinese females, Chinese males, US females, and US males, respectively. Chinese females therefore had the lowest prevalence of both cigarette smoking and binge drinking, while Chinese boys had a lower prevalence of binge drinking compared to both US boys and girls. 

The intervention did not generate statistically significant (tailed alpha = 0.05) effects on CIU, and prevalence of cigarette smoking and binge drinking. Additional analysis also failed to detect any statistically significant interaction between the intervention and the focused relationships between the three addictive behaviors. Thus, reported below were the results from the analyses involving subjects in both “program” and “control” conditions, with intervention status adjusted as a covariate.

**Figure 1:**
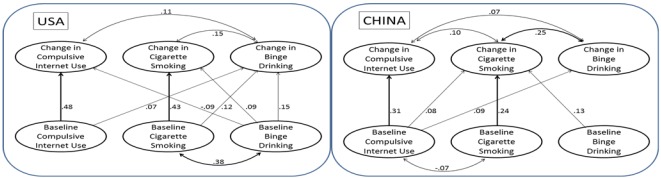
Female students only: Concurrent and longitudinal predictive relationships among CIU, cigarette smoking, and binge drinking.

**Figure 2:**
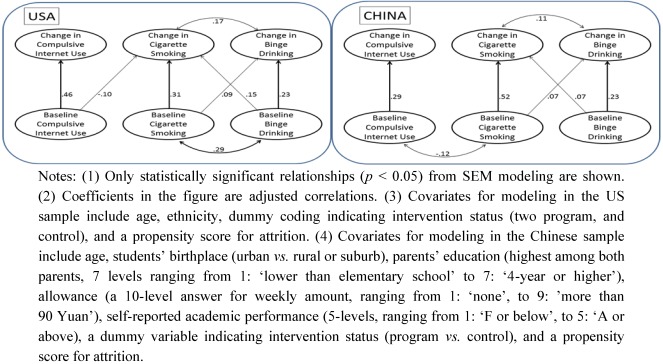
Male students only: Concurrent and longitudinal predictive relationships among CIU, cigarette smoking, and binge drinking.

The concurrent and predictive relationships between CIU, cigarette smoking, and binge drinking are depicted separately for females (Figure 1) and males (Figure 2) since results were found to vary by gender. Two pathway diagrams are included side-by-side in each figure, with the left diagram revealing findings among the US youth, and the right for the Chinese. Chi-square tests for the unrestricted and fitted models indicate a good model fit for all four models (dof = 80, χ^2^ > 500 for the models of US females and US males; dof = 75, χ^2^ > 4,000 for the models of Chinese females and Chinese males). In all figures, only statistically significant (*p* < 0.05) relationships are illustrated, with the curved double-headed arrows depicting concurrent relationships, and straight single-headed arrows depicting predictive relationships. Four major findings emerged from the pattern of relationships.

First, data from both studies revealed the lack of positive cross-sectional relationships between CIU and substance use. Differences between US and Chinese youth were found in the relationships between baseline measures of CIU, cigarette use, and binge drinking. Baseline CIU was negatively correlated with baseline cigarette smoking among Chinese female and male youth (r = −0.07 and −0.12, respectively), but not among US female or male youth.

Second, longitudinally, higher baseline CIU predicted elevated substance use in female students, but not in male students. Among females, higher baseline CIU levels resulted in more binge drinking at 1-year follow-up among US students (r = 0.07), and in more cigarette smoking (r = 0.08) and more binge drinking at 1-year follow-up (r = 0.09) among Chinese students. However, among males, the only predictive relationship between CIU and substance use was that among US males, in which higher baseline CIU levels resulted in less cigarette smoking at 1-year follow-up (r = −0.10). 

Third, change in CIU was positively related with change in substance use among females, but not males. More specifically, change in CIU was positively related with change in binge drinking among both US (r = 0.11) and Chinese (r = 0.07) female students, and with change in cigarette smoking among Chinese female students (r = 0.10) only. However, none of the relationships between concurrent changes were statistically significant among male students.

Fourth, baseline substance use did not predict the increase in CIU over 1 year. Regarding cigarette use and binge drinking among female and male US and Chinese students, the only statistical finding was that baseline binge drinking predicted a reduction in CIU over 1 year among the US female students (r = −0.09). 

Overall, a complete pattern of positive relationships emerged between cigarette smoking and binge drinking among US youth. The patterns are referred to as complete because there were significant relationships between all possible pathways. Concurrently, baseline cigarette smoking and binge drinking were found to be positively correlated (r = 0.38 in US females, and r = 0.29 in US males), as were their change scores a year later (r = 0.15 in US females, and r = 0.17 in US males). Predictively, baseline cigarette smoking predicted change in binge drinking (r = 0.12 and 0.09 in US female and males, respectively); while baseline binge drinking also predicted change in cigarette smoking (r = 0.09 and 0.15 in US female and males, respectively). In comparison, the pattern of relationships between cigarette smoking and binge drinking were less complete among Chinese youth, and they were the least complete between CIU and any of the substance use measures.

## 6. Discussion

This study documents both the concurrent and predictive longitudinal relationships among CIU, cigarette smoking, and binge drinking among non-traditional high school students in the US and China. Viewing CIU as a process addiction behavior, and cigarette smoking and binge drinking as substance use addiction behaviors, the findings help document the relationships between a process addiction and two substance use addictions, with the relationships between the two types of substance use addiction serving as a reference for comparison. 

Five major findings can be summarized from the data: (1) CIU was not positively related with substance use at baseline. (2) There was a positive predictive relationship between baseline CIU and change in substance use among female, but not male students. (3) Relationships between concurrent changes in CIU and substance use were also found among female, but not male students. (4) Baseline substance use did not predict an increase in CIU from baseline to 1-year follow-up. (5) A complete reciprocally predictive relationship was found among US females and males for the relationship between cigarette smoking and binge drinking; but no reciprocally predictive relationship was found between CIU and either type of substance use among either US or Chinese youth. 

While the relationships are complex, these unique findings can be applied to test theoretical models of addiction. For example, a Syndrome Model of Addiction suggests that substance addiction and process addiction are all specific expressions of underlying shared manifestations and sequelae for a general addiction syndrome [18]. Guided by the syndrome hypothesis, different manifestations (addiction types) of an underlying addiction syndrome may be correlated, and the change in one addiction manifestation should co-vary (concurrent or substitute manifestations) with the change in another manifestation. Our findings regarding cigarette smoking and binge drinking among US youth shows a strong pattern of concurrent manifestations of addiction, and is thus consistent with the syndrome model. However, due to the overall pattern of results showing the lack of any cross-sectional, predictive, and concurrent relationships between CIU and substance use among male students, our data offers evidence that is inconsistent with the Syndrome Model of Addiction between CIU and substance use among male students from either the US or China. The relationship between CIU and cigarette smoking among Chinese females may indicate a certain level of substitute addiction occurring between CIU and cigarette use. Chinese females with a higher “general” addiction level may vacillate between CIU or cigarette smoking, thereby resulting in a small negative correlation between the two behaviors at baseline. However, because of the “general” addiction tendency, females with higher CIU levels at baseline may also have a higher likelihood of smoking at one-year follow-up.

The “PACE” model for addiction hypothesizes that Pragmatics, Attraction, Expectations, and Communication are the etiologic elements that explain a person’s addiction to an array of addiction behaviors or to a specific addictive behavior [39]. While consistent with the syndrome model of addiction in that shared factors may cause an array of addictions, the PACE model emphasizes the fact that people with addiction syndrome do not randomly choose one or an array of addictions, or alternate from one to another. While there are shared biological and environmental factors behind the concurrent manifestations of addiction, the specific expressions of addiction may be studied in the framework of the PACE model involving pathways that link various PACE elements [39]. Guided by the PACE model, the lack of overall correlation between CIU and substance use (in reference to the comprehensive correlations between smoking and binge drinking) may indicate the need to study the specific risk factors and pathways for CIU and use of other substances. For example, the availability of supply and acquisition skills (Pragmatics) may be different; the appetitive effects (Attraction) may also be different; taken together with the synergy of preferentially socializing with different sub-groups of students (Communication), the Expectations and therefore the actual adoption of internet or substance addiction may be different. 

With regard to the positive associations between baseline CIU and change in substance use, and between change in CIU and change in substance use, the positive associations are detected among female, but not male students. Previous studies have reported a trend of positive associations between internet addiction and risk factors for substance use or substance use dependencies [10,11,12,13], although none of them conducted analyses separately by gender. Thus, our finding indicates that the association between CIU and substance use may differ by gender. This is consistent with other research that has identified gender as an important factor that moderates the neurobiological, cognitive, and psychosocial mechanisms for both cigarette use [40] and binge drinking [41]. In general teenage males binge drink more frequently than females, and teen binge drinking is associated with a variety of problem prone behaviors [42]. Binge drinking may reflect a greater masculinity-based gender role orientation, related to the externalization of behaviors [43]. Therefore, males who engage in CIU may not be any more likely to binge drink in the future compared to males who do not engage in CIU and are otherwise similar in levels of masculinity. Females who engage in CIU, on the other hand, may be more likely to demonstrate masculine gender roles (e.g., if they are online game players), and hence, be more likely to binge drink in the future compared to other females who are higher in femininity. This and other explanations of CIU as a predictor of prospective binge drinking among females in both countries should be examined in future studies.

In addition, previous studies have revealed a possible difference in types of CIU by gender. Huang *et al.* [32] reported that the most prevalent type of internet use differed for Chinese female and male youth, with females being more likely to use online social networking sites, and males being more likely to use online gaming programs. Future studies would be needed to reveal the mechanisms by which the associations between CIU and substance use differ by gender, as well as examine the types of internet use, and their specific roles in the addictive behaviors of females and males. 

One possible explanation for the differences found among US and Chinese youth is that the same behavior may be perceived differently in different cultures, in addition to being different between females and males. For example, binge drinking by youth at a social gathering may not be considered deviant behavior in China, while any youth alcohol consumption is prohibited by laws in the US. Further, both the prevalence and relationships between cigarette smoking and binge drinking were found to differ between the US and Chinese samples, and these differences may reflect the differential meaning of cigarette smoking and binge drinking across race and gender. The vast differences in smoking prevalence, and reasons for smoking, among male and female youth in China have been previously documented [44]. In addition, internet use and its addiction may also bear different meanings and consequences between US and Chinese youth. For example, internet access in cyber cafés is much more common in China than in the US; therefore, the relationship between CIU and substance use among Chinese youth may be confounded by the physical and social settings for internet use. Thus, our finding supports the notion that the overall cultural context plays an important role in studying the current and lifetime co-morbidity and prevention of addictive behaviors. 

Findings from previous cross-sectional studies have revealed that CIU is positively correlated with substance use [23,24], and has similar relationships with some common risk factors for substance use (e.g., hostility, depression [10], family factors [45]). However, in this study, baseline CIU was not related to baseline substance use among US youth, and was negatively correlated with baseline cigarette smoking among Chinese youth. This difference in findings by country may stem from the uniqueness of the samples, study design, or measures used. Nevertheless, our finding is salient because it offers evidence for a substitute model of addiction, such as the syndrome model or “PACE” model for addictions. For example, due to there being a common risk factor profile for an general level of addiction, a person may have a higher tendency to indulge in one or more addictive behaviors in a specific time period and environment; however, the addictive behavior may switch from one to another along with the change in time period or environment. Thus, although CIU may share common risk factors with substance use, CIU as a behavior may not be correlated, or even may be negatively correlated with substance use depending upon the contextual factors.

Results of this study show that prospectively, baseline substance use reduces CIU among US youth when assessed one year later, but no significant relationship exists between baseline substance use and change in CIU among Chinese youth. Reciprocally, baseline CIU increases substance use among Chinese females, yet reduces cigarette smoking among US males. While the data revealed a small prospective effect between CIU and substance use, the data did not suggest clear and substantively significant directionality in the pattern of relationships. One possible explanation for this is that the effects of CIU and the effect on CIU may be closely coupled with the particular type of internet use in which youth frequently engage. An alternative hypothesis proposed for further study is that addiction to particular types of websites may increase substance use, or that particular types of CIU may be promoted by substance use. 

This study draws data from two longitudinal trials with both CIU and substance use behaviors assessed simultaneously at baseline and one-year follow-up. Thus, the data offered an unprecedented opportunity to examine the concurrent and predictive relationships between CIU and substance use behaviors, and differences by country and gender. 

Nevertheless, the study has inherent weaknesses. The first weakness is that the studies from which the data were drawn were not designed to focus on CIU. The assessment of IA needed to be shortened to focus on the compulsive use dimension, which may not be representative of the full spectrum of IA behavior [7]. Our measure of IA is thus labeled as Compulsive Internet Use (CIU), which was an un-validated sub-scale adapted from a 4-dimensional measure of Problematic Internet Use suggested by Davis *et al.* [33]. Also, we are not aware of any study that has reported on the validity of the Davis “Problematic Internet Use” scale among high-risk US or Chinese youth. Therefore, unique findings from this report between CIU and substance use may not be generalized to validate or invalidate other findings where IA was assessed differently. The second weakness could be due to the two studies being conducted in US and China, separately. Our aim was to compare the results from data drawn from two countries, but the comparability may be compromised by several major differences in the two studies. First, although youth in both studies were considered “higher risk” high school students enrolled in non-regular high schools, the US students were enrolled in “Continuation High Schools” often due to behavioral problems, while the Chinese students were enrolled in “Vocational High Schools” mainly due to academic or economic reasons. Second, although the CIU items were coordinated to be examined in the same way, the overall study designs and other measures were not exactly the same. Thus, the cross-country comparison of findings may be informative, but we are not able to conclude that the differences in findings are attributable to the differences between the Eastern and Western cultural context. Regarding the study samples, there are both strengths and weaknesses that stem from this study being conducted among high-risk youth. The strengths are due to the samples having a higher prevalence of addictive behaviors, which thereby allows for greater statistical power for the study, and yields results that are directly applicable to the needs of the sub-group of youth who need the most help. Conversely, a weakness is that the conclusions may not be extended to all youth, most of whom attend regular academic schools, and are at lower-risk for substance use.

In summary, the harmful effects of Internet Addiction are starting to be recognized in the media and the research field, yet the scientific understanding of the behavior is still in its infancy. The findings from this study suggest that the etiologic pathway to Internet Addiction may share common aspects with the pathways for substance use, although the unique aspects of Internet Addiction warrant clearer examination in future studies. 
